# Optical coherence tomography biomarkers as outcome predictors to guide dexamethasone implant use in patients with iERM: a randomized controlled trial

**DOI:** 10.1186/s12886-024-03429-2

**Published:** 2024-04-25

**Authors:** Rong Luan, Manqiao Wang, Yi Gong, Boshi Liu, Xinyuan Huang, Jie Wang, Shuo Sun, Jinzhi Zhao, Xiteng Chen, Qianhui Yang, Juping Liu, Yan Shao, Xiaorong Li

**Affiliations:** Tianjin Key Laboratory of Retinal Functions and Diseases, Tianjin Branch of National Clinical Research Center for Ocular Disease, Eye Institute and School of Optometry, Tianjin Medical University Eye Hospital, 384300 Tianjin, China

**Keywords:** Epiretinal membrane, Intravitreal dexamethasone, Vitrectomy, OCT, Hyperreflective foci, Vitreous fluid, Cytokine

## Abstract

**Background:**

We aimed to investigate the anatomical features of optical coherence tomography (OCT) and vitreous cytokine levels as predictors of outcomes of combined phacovitrectomy with intravitreal dexamethasone (DEX) implants for idiopathic epiretinal membrane (iERM) treatment.

**Methods:**

A prospective, single-masked, randomized, controlled clinical trial included 48 eyes. They were randomly assigned in a 1:1 ratio to undergo the DEX group (combined phacovitrectomy with ERM peeling and Ozurdex implantation) and control group (phacovitrectomy only). Best-corrected visual acuity (BCVA) and central macular thickness (CMT) were assessed at 1 d, 1 week, 1 month, and 3 months. The structural features of OCT before surgery were analysed for stratified analysis. Baseline soluble CD14 (sCD14) and sCD163 levels in the vitreous fluid were measured using ELISA.

**Results:**

BCVA and CMT were not significantly different in the DEX and control groups. Eyes with hyperreflective foci (HRF) at baseline achieved better BCVA (*P*_time*group_=0.746; *P*_group_=0.043, Wald χ²=7.869) and lower CMT (Ptime*group = 0.079; Pgroup = 0.001, Wald χ²=6.774) responses to DEX during follow-up. In all patients, the mean vitreous level of sCD163 in eyes with HRF was significantly higher than that in eyes without HRF (*P* = 0.036, Z=-2.093) at baseline. In the DEX group, higher sCD163 predicted greater reduction in CMT from baseline to 1 month (*r* = 0.470, *P* = 0.049).

**Conclusions:**

We found that intraoperative DEX implantation did not have beneficial effects on BCVA and CMT over a 3-month period in all patients with iERM, implying that the use of DEX for all iERM is not recommended. In contrast, for those with HRF on OCT responded better to DEX implants at the 3-month follow-up and thier vitreous fluid expressed higher levels of sCD163 at baseline. These data support the hypothesis that DEX implants may be particularly effective in treating cases where ERM is secondary to inflammation.

**Trial registration:**

The trail has been registered at Chinese Clinical Trail Registry(https://www.chictr.org.cn) on 2021/03/12 (ChiCTR2100044228). And all patients in the article were enrolled after registration.

**Supplementary Information:**

The online version contains supplementary material available at 10.1186/s12886-024-03429-2.

## Introduction

Idiopathic epiretinal membrane (iERM) is a common disease with no specific cause. Avascular membranes can distort the retina and blood vessels of the macular area, causing deterioration of central vision, visual distortion, and monocular diplopia [1]. Currently, medication therapy is ineffective for iERM. Pars plana vitrectomy (PPV) combined with ERM peeling surgery is the most effective treatment for iERM to improve best-corrected visual acuity (BCVA) [2]. However, a considerable proportion of patients do not respond satisfactorily to vitrectomy. Persistent macular edema (ME) is a common cause of poor visual outcomes after PPV for ERM removal [3].

Dexamethasone (DEX) is a corticosteroid with a high hydrophilicity that facilitates a high vitreous concentration. Furthermore, it is five times more potent than triamcinolone [4]. However, its clinical utility is limied by its short half-life. This has necessitated the development of a biodegradable DEX drug delivery system. The slow-release DEX preparation, Ozurdex, is a 6-mm implant containing 700 mg of DEX in a biodegradable polymer (Novadur, Allergan, Irvine, CA, USA). Ozurdex has been successfully applied for the treatment of diabetic ME, retinal vein occlusion, and noninfectious posterior uveitis, and has also been used off-label for treatment of refractory cystoid macular edema (CME) in vitrectomised eyes [5].

Experimentally, corticosteroids can potentially inhibit inflammation without evidence of demonstrable retinal toxicity. Results [6]–[10] regarding the efficacy of DEX for accelerating central macular thickness (CMT) reduction and BCVA improvement in iERM vary among different clinical studies. Previous studies have drawn controversial conclusions, which may indicate that the prognosis of DEX treatment is related to patient characteristics. Therefore, individualised treatment is critical for optimal functional outcomes and disease management.

Anatomical measures on optical coherence tomography (OCT) include the precise evaluation of retinal thickness, and various microstructural abnormalities such as vitreous changes, hyperreflective foci (HRF), and retinal vasculature [11]. Previous studies have examined the predictive value of baseline OCT measures in patients with iERM [12]–[15]. We hypothesised that the distinct structural changes identified on OCT could be used as predictors of treatment responses to DEX implants. The purpose of this study was to assess the potential anatomical and visual outcomes of a 0.7-mg slow-release preparation of DEX administered during vitrectomy surgery.The correlation between characteristics identified on OCT and the expression of cytokines in the vitreous was assessed, allowing for the identification of biomarkers that could predict the treatment response to DEX implants after ERM removal surgery.

## Methods

This was a prospective, single-masked, randomized, comparative study. All procedures performed in the study involving human participants were in accordance with the Declaration of Helsinki and the ethical standards of the Ethics Committee of Tianjin Medical University Eye Hospital. This study has been approved by the Ethics Committee of Tianjin Medical University Eye Hospital (2020KY-34) and has been registered in Chinese Clinical Trail Registry (https://www.chictr.org.cn) (ChiCTR2100044228).

### Patients and clinical examination

This study enrolled patients diagnosed with iERM combined with cataracts who were scheduled to undergo phacovitrectomy combined with ERM peeling at Tianjin Medical University Eye Hospital, Tianjin, China, between July 2021 and December 2022. The patients were randomly assigned in a 1:1 ratio by drawing lots.: those receiving the combined 27-G phacovitrectomy with ERM peeling plus DEX implants (DEX group), and those undergoing 27-G PPV phacovitrectomy ERM peeling alone (control group). Allocation was performed before the operation, and all patients were masked to the assignment. The identified information was provided to the surgeons and the researchers before surgery but remained inaccessible to the patients.

Patients were included in the analysis if they fulfilled the following criteria: (1) age > 40 years (2), diagnosis of iERM requiring vitrectomy, and (3) confirmed visual function and OCT findings after vitrectomy during follow-up. For patients who underwent bilateral vitrectomy treatment, both eyes were included. The exclusion criteria were as follows: (1) other concomitant ocular diseases that cause macular morphology and visual function (i.e., diabetic retinopathy, neovascular age-related macular degeneration, or choroidal neovascularization resulting from various causes, retinal vein occlusion, uveitis, and recent intraocular surgery, which may potentially lead to postsurgical ME); (2) other ocular conditions that seriously affect VA (i.e., high myopia) except for cataracts; and (3) treatment with systemic or intraocular corticosteroids within 6 months preoperatively.None of the patients had ocular or retinal diseases, except for mild refractive error or cataracts that did not affect the BCVA.

Follow-up visits were scheduled at 1 d, 1 week, 1 month, and 3 months after surgery and included BCVA reported in the Snellen fraction with minimum angle of resolution (logMAR) conversion for statistical analysis, intraocular pressure (IOP) measurement, slit-lamp and fundus examination, and OCT analysis [16] (VG200; SVision Imaging, Ltd., Luoyang, China).

OCT images were classified according to the following baseline parameters (1). CMT: Retinal thickness was obtained using automatic thickness measurements. The variation ratio of CMT (△CMT) was defined as CMT_pr_ - CMT_Po_, where CMT_pr_ and CMT_Po_ are CMT before and after surgery, respectively (2). CME: presence of cystoid changes (3). Microcysts ME (MME): degenerative cysts. Presenting with presence of isolated parafovea inner nuclear layer (INL) cystoid change (4). Presence of hyperreflective foci (HRF) (5). Ectopic inner foveal layer (EIFL): Presence of ectopic inner foveal layers extending continuously from the INL and IPL across the central fovea [17] (6). Disorganization of retinal inner layers (DRIL): the boundaries between the ganglion cell-inner plexiform layer complex and INL, as well as between the INL and outer plexiform layer were assessed within the central 1000 μm (7). Continuity of the inner segment/outer segment (IS/OS) layer. All OCT biomarkers are shown in Supplement Fig. 1.

### Outcomes

The primary outcome measure included the postoperative BCVA and CMT. Meanwhile, the secondary outcome measures was the postoperative IOP.

### Surgical procedure

All surgeries were performed by the same surgeon using the Constellation Vitrectomy System (Alcon, USA) for a standard three-port sutureless phacovitrectomy combined with ERM peeling. The ERM was peeled with intraocular forceps after application of 0.1 ml of 0.25% brilliant blue G coloration for approximately 1 min. In both groups, the ILM was routinely removed. In the DEX group, a 0.7-mg sustained-release DEX (Ozurdex®) was carefully injected through the superotemporal scleral port applying gentle force gradually to avoid any damage to the retina. The position of the implant was confirmed at the end of the procedure using an indirect operating microscope. During the preoperative period, patients in both groups were treated using topical antibiotics (levofloxacin 1.5%) four times per day for 3 days. Whereas during the postoperative period, both patients were treated using levofloxacin 1.5% and prednisolone acetate 1% four times per day for 7 days to taper down weekly in 1 month.

### Vitreous fluid collection and laboratory assessments

Undiluted vitreous samples (approximately 0.5 mL) were obtained at the onset of vitrectomy by aspirating them into a 1-mL syringe connected to the vitreous cutter before initiating intravitreal infusion of balanced salt solution. The collected samples were then transferred to a tube, promptly placed in liquid nitrogen, and frozen at -80 °C until assayed.

In these samples, sCD14 and sCD163 levels were quantified using enzyme-linked immunosorbent assay (ELISA) with reagents provided by Thermo Fisher Scientific Inc., Waltham, MA, USA). The lower limits of detection for sCD14 and sCD163 were 6 and 30 pg/mL, respectively.

### Statistical analysis

The sample size was calculated based on the primary outcome measures with two-sided significance α = 0.05 and power = 0.8. Data analysis was performed using SPSS statistical software (SPSS Inc., Chicago, IL, USA). The values are presented as mean (standard deviation [SD]). An independent-samples *t*-test was conducted to compare continuous variables between groups. The Mann–Whitney-U test was used for non-parametric analysis. An *χ*^*2*^ test was used to compare dichotomous variables between groups.

To account for the correlated nature of our data, we employed the generalised estimating equation (GEE) procedure. We calculated the between-group differences in functional treatment response and OCT baseline measures using a GEE model, with baseline BCVA included as a covariate. Similarly, we calculated the differences in anatomical outcomes and included baseline CMT as a covariate. The GEE model was also used to determine whether the retinal microstructure at baseline determined outcomes during follow-up. Bonferroni’s correction was used for post hoc pairwise comparisons. Outcome measures were assessed by stratifying the analysis of the predictors at baseline, including: (1) CMT (2), CME (3), MME (4), HRF (5), EIFL (6), DRIL, and (7) IS/OS continuity. Measurement data are presented as mean (SD). A *P* value < 0.05 was considered statistically significant.

The significance of differences in sCD163 and sCD14 levels among the different groups was analysed using the Mann–Whitney test. To determine there was a significant correlation between sCD163 and △CMT, Spearman correlation tests were employed. A two-tailed *P* value < 0.05 was considered statistically significant.

## Results

### Baseline characteristics

Forty-eight subjects (48 eyes) with iERM were included in this study between July 2021 and December 2022., of which 24 eyes underwent 27-G phacovitrectomy with ERM peeling only, and the other underwent 27-G phacovitrectomy with ERM peeling plus DEX implants. Five patients ( in the DEX group) were lost to follow-up. The flow diagram is shown in Supplement Fig. 2. All procedures were uneventful in both groups. There were no cases of capsule rupture, zonular dialysis, or dropped lens material.

Baseline non-ocular and ocular characteristics are summarised in Supplement Table 1. Supplement Table 1 compares the sex and mean age of the groups. Notably, there was a higher proportion of females in the sexagenarian age category in both groups compared with that of the other age categories.

BCVA and OCT baseline characteristics are shown in Supplement Table 1. The median presenting BCVAs were 0.58 ± 0.25 and 0.57 ± 0.29 (logMAR) in the adjunct and control groups, respectively. CMT did not differ significantly between the groups (444.8 ± 109.0 and 449.0 ± 82.8, *P* = 0.891). Mean IOP readings were 13.90 ± 0.74 mmHg and 14.97 ± 0.85 mmHg in the DEX and control groups.

Furthermore, there were no significant between-group differences in preoperative OCT baseline characteristics, including macular cysts (*P* = 0.515), MME (*P* = 0.613), HRF (*P* = 0.977), EIFL(*P* = 0.864), DRIL (*P* = 0.515), and IS/OS continuity (*P* = 0.836). There were no differences in baseline ocular characteristics between the groups (Supplement Table 1).

### Functional and anatomical outcome

Table 1 shows the postoperative BCVA and CMT values in the two groups. Between-group comparison of the two parameters (while controlling for the effects of the preoperative values, BCVA (logMAR), CMT, and IOP) showed no significant differences during follow-up (*P* = 0.391, *P* = 0.315, and *P* = 0.594, respectively by GEE).

There was no significant difference in BCVA between the two groups at 1 d.

(*P* = 0.844, Wald *χ²*=0.04), 1 week (*P* = 0.546, Wald *χ²*=0.37), 1 month (*P* = 0.275, Wald *χ²*=1.19), or 3 months (*P* = 0.250, Wald *χ²*=1.32) after surgery.

Furthermore, no significant difference was observed in CMT between the two groups at 1 d (*P* = 0.950, Wald *χ²*=0.004), 1 week (*P* = 0.139, Wald *χ²*=2.19), 1 month (*P* = 0.337, Wald *χ²*=0.923), or 3 months (*P* = 0.367, Wald *χ²*=0.812) after surgery.

Regarding the IOP, there was no significant difference in IOP between the two groups at 1 d (*P* = 0.528, Wald *χ²*=0.40), 1 week (*P* = 0.804, Wald *χ²*=0.06), 1 month (*P* = 0.938, Wald *χ²*=0.01), or 3 months (*P* = 0.614, Wald *χ²*=0.26) after surgery.

**Table 1:** Postoperative values of BVCA and CMT

			DEX group	Wald *χ²* Value	*P*_group*time_	*P*_time_	*P*_group_
BCVA (logMAR, Snellen), Mean (SD)	1 day	0.492 ± 0.048	0.478 ± 0.053	0.813	0.846	**< 0.001**	0.391
	1 week	0.365 ± 0.045	0.328 ± 0.042				
	1 month	0.301 ± 0.047	0.234 ± 0.040				
	3 months	0.261 ± 0.036	0.200 ± 0.044				
*P*_time_		**< 0.001**	**< 0.001**				
CMT (µm), Mean (SD)	1 day	431.97 ± 14.09	430.43 ± 14.11	3.814	0.282	**< 0.001**	0.315
	1 week	403.36 ± 18.45	364.75 ± 18.63				
	1 month	376.19 ± 20.09	347.03 ± 22.86				
	3 months	362.44 ± 17.99	338.65 ± 19.41				
*P*_time_		**< 0.001**	**< 0.001**				
IOP (mmHg) Mean (SD)	1 day	13.90 ± 0.74	14.97 ± 0.85	4.28	0.930	0.547	0.594
	1 week	14.43 ± 0.49	15.06 ± 0.85				
	1 month	14.97 ± 1.09	15.50 ± 0.69				
	3 months	14.07 ± 0.92	14.92 ± 0.68				
*P*_time_		0.497	0.928				

BCVA = best corrected visual acuity; CMT = center macular thickness; IOP = intraocular pressure; SD = standard deviation. * Indicates a statistically significant difference between two groups in post hoc analysis

### Optical coherence tomography predictors for treatment response

The value of all OCT measures for prediction of BCVA and CMT changes in response to DEX are shown in Tables 2 and 3.

Patients who presented with HRF at baseline were more likely to have better BCVA (*P*_time*group_=0.746; *P*_group_=0.043, Wald *χ²*=7.869) and lower CMT (*P*_time*group_=0.079; *P*_group_=0.001, Wald *χ²*=6.774) in response to DEX. In the two-by-two comparison, eyes with HRF had significantly better visual acuity in the DEX group at 1 month (Wald *χ²*=3.95, *P* = 0.047) and 3 months (Wald *χ²*=6.91, *P* = 0.009) postoperatively, and lower CMT at 1 week (Wald *χ²*=7.17, *P* = 0.007), 1 month (Wald *χ²*=16.78, *P* < 0.001), and 3 months (Wald *χ²*=7.83, *P* = 0.005). CME, MME, DRIL, EIFL, continuity of the IS/OS layers, and CMT at baseline did not correlate with visual and anatomical outcomes after DEX implantation.

**Table 2:** Baseline predictors of best-corrected visual acuity outcome after surgery

	Control Group				DEX Group				P_time*group_	P_time_	P_group_
	1 day	1 week	1 month	3 months	1 day	1 week	1 month	3 months			
**Preoperative CMT**											
<400 μm	0.301 ± 0.035	0.244 ± 0.039	0.199 ± 0.048	0.192 ± 0.038	0.402 ± 0.058	0.209 ± 0.036	0.165 ± 0.041	0.108 ± 0.042	0.253	**< 0.001**	0.767
≥400 μm	0.620 ± 0.070	0.457 ± 0.068	0.379 ± 0.069	0.315 ± 0.053	0.561 ± 0.090	0.450 ± 0.066	0.300 ± 0.064	0.288 ± 0.075	0.486	**< 0.001**	0.619
**CME**											
Absent	0.396 ± 0.049	0.298 ± 0.042	0.234 ± 0.046	0.208 ± 0.042	0.423 ± 0.056	0.270 ± 0.037	0.172 ± 0.032	0.139 ± 0.037	0.649	**< 0.001**	0.498
Present	0.706 ± 0.079	0.522 ± 0.093	0.459 ± 0.100	0.384 ± 0.072	0.798 ± 0.137	0.638 ± 0.126	0.565 ± 0.072	0.482 ± 0.132	0.977	**0.001**	0.428
**MME**											
Absent	0.345 ± 0.037	0.263 ± 0.035	0.199 ± 0.044	0.183 ± 0.037	0.383 ± 0.060	0.289 ± 0.048	0.188 ± 0.036	0.135 ± 0.049	0.438	**< 0.001**	0.936
Present	0.708 ± 0.084	0.538 ± 0.081	0.458 ± 0.078	0.377 ± 0.055	0.620 ± 0.086	0.381 ± 0.076	0.295 ± 0.073	0.267 ± 0.074	0.547	**< 0.001**	0.168
**HRF**											
Absent	0.417 ± 0.046	0.311 ± 0.044	0.246 ± 0.044	0.227 ± 0.039	0.467 ±0.081	0.349 ± 0.060	0.277 ± 0.052	0.271 ± 0.060	0.981	**< 0.001**	0.552
Present	0.602 ± 0.098	0.452 ± 0.090	0.389 ± 0.096	0.313 ± 0.068	0.500 ± 0.061	0.301 ± 0.052	0.176 ± 0.048^*^	0.104 ± 0.040^*^	**0.746**	**< 0.001**	**0.043**
**EIFL**											
Absent	0.389 ± 0.044	0.321 ± 0.044	0.275 ± 0.044	0.264 ± 0.042	0.409 ± 0.062	0.289 ± 0.053	0.190 ± 0.046	0.165 ± 0.050	0.366	**< 0.001**	0.407
Present	0.722 ± 0.109	0.472 ± 0.110	0.362 ± 0.118	0.253 ± 0.058	0.641 ± 0.089	0.411 ± 0.063	0.328 ± 0.072	0.260 ± 0.091	0.862	**< 0.001**	0.701
**DRIL**											
Absent	0.357 ± 0.048	0.284 ± 0.041	0.213 ± 0.041	0.201 ± 0.032	0.341 ± 0.048	0.227 ± 0.043	0.170 ± 0.051	0.136 ± 0.056	0.849	**< 0.001**	0.377
Present	0.693 ± 0.080	0.492 ± 0.089	0.439 ± 0.061	0.353 ± 0.072	0.637 ± 0.082	0.446 ± 0.061	0.310 ± 0.056	0.271 ± 0.065	0.701	**< 0.001**	0.377
**IS-OS continuity**											
continuous	0.446 ± 0.057	0.356 ± 0.051	0.310 ± 0.049	0.256 ± 0.035	0.405 ± 0.056	0.268 ± 0.040	0.170 ± 0.041	0.138 ± 0.046	0.521	**< 0.001**	0.081
disrupted	0.685 ± 0.055	0.425 ± 0.041	0.291 ± 0.043	0.305 ± 0.043	0.709 ± 0.082	0.514 ± 0.062	0.434 ± 0.025	0.372 ± 0.078	0.327	**< 0.001**	0.123

**Table 3:** Baseline predictors of central macular thickness outcome after surgery

	Control Group				DEX Group				P_time*group_	P_time_	P_group_
	1 day	1 week	1 month	3 months	1 day	1 week	1 month	3 months			
<400 μm	331.44 ± 26.98	318.96 ± 33.51	322.06 ± 35.01	303.06 ± 25.65	352.73 ± 25.66	277.47 ± 16.29	278.03 ± 26.69	271.36 ± 23.62	0.050	**< 0.001**	0.526
≥400 μm	505.69 ± 14.90	467.58 ± 22.39	418.80 ± 23.96	408.80 ± 24.36	496.08 ± 17.32	438.97 ± 32.34	402.97 ± 38.90	393.18 ± 31.95	0.613	**< 0.001**	0.885
**CME**											
Absent	436.59 ± 17.67	411.53 ± 20.98	392.70 ± 20.31	368.41 ± 16.43	421.26 ± 15.29	372.96 ± 14.19	344.96 ± 19.30	339.51 ± 16.28	0.188	**< 0.001**	0.136
Present	428.73 ± 17.10	389.87 ± 32.62	342.45 ± 41.01	354.30 ± 45.60	455.29 ± 32.27	306.96 ± 65.93	340.93 ± 94.21	317.96 ± 77.57	**< 0.001**	**< 0.001**	0.753
**MME**											
Absent	400.96 ± 19.97	380.79 ± 24.92	369.06 ± 23.61	350.39 ± 18.64	418.89 ± 20.17	363.89 ± 16.35	340.11 ± 23.12	328.40 ± 17.51	0.237	**< 0.001**	0.609
Present	471.26 ± 16.29	428.26 ± 26.38	375.37 ± 34.35	369.82 ± 35.00	456.04 ± 18.57	376.06 ± 32.56	364.39 ± 38.33	358.94 ± 33.42	0.082	**< 0.001**	0.571
**HRF**											
Absent	385.44 ± 19.23	352.77 ± 26.99	335.69 ± 31.79	321.34 ± 25.74	423.82 ± 22.25	353.52 ± 28.74	363.42 ± 36.87	350.96 ± 28.43	0.591	**< 0.001**	0.501
Present	485.65 ± 14.72	460.95 ± 16.71	419.65 ± 13.63	406.75 ± 17.96	450.37 ± 391.38	391.38 ± 19.60^*^	339.13 ± 14.99^*^	335.88 ± 18.81^*^	0.079	**< 0.001**	**0.001**
**EIFL**											
Absent	403.47 ± 15.06	363.87 ± 22.61	353.98 ± 23.26	334.87 ± 18.93	395.52 ± 20.01	339.85 ± 14.22	335.35 ± 23.68	322.93 ± 19.14	0.827	**< 0.001**	0.524
Present	523.81 ± 22.11	493.38 ± 30.84	424.24 ± 38.20	423.52 ± 41.61	499.44 ± 16.81	420.10 ± 49.20	375.94 ± 46.97	360.70 ± 46.16	0.561	**< 0.001**	0.308
**DRIL**											
Absent	426.18 ± 20.22	393.79 ± 25.63	377.92 ± 25.44	357.26 ± 19.90	417.82 ± 26.00	333.08 ± 28.34	316.19 ± 31.45	313.53 ± 25.86	0.211	**< 0.001**	0.167
Present	436.35 ± 16.70	410.58 ± 24.64	364.58 ± 31.64	362.35 ± 31.11	451.89 ± 13.97	403.00 ± 19.19	384.45 ± 28.67	370.10 ± 24.92	0.479	**< 0.001**	0.778
**IS-OS continuity**											
continuous	448.00 ± 18.48	422.22 ± 25.74	376.22 ± 29.94	374.00 ± 32.70	439.98 ± 11.43	391.09 ± 13.92	372.53 ± 22.53	357.69 ± 20.57	0.345	**< 0.001**	0.106
disrupted	451.47 ± 31.08	405.67 ± 47.95	333.87 ± 51.58	321.07 ± 46.22	483.91 ± 33.54	355.6 ± 663.43	366.91 ± 81.06	340.91 ± 68.79	**< 0.001**	**< 0.001**	0.912

### Baseline differences in vitreous concentrations of sCD163 and sCD14 in eyes with and without HRF

**Fig. 1:**
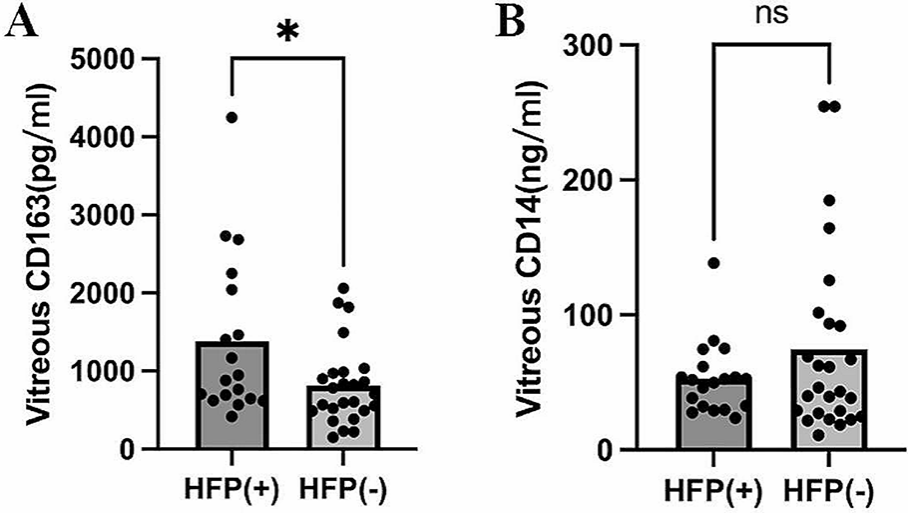
Intravitreous concentrations of sCD163 and sCD14 in eyes with and without HRF. (**A**) Intravitreous concentration sCD163 in eyes with or without HFP. *P = 0.036, *Z*=-2.09. (**B**) Intravitreous concentration of sCD14 in eyes with or without HRF. ns*P* = 0.313, *Z*=-1.01

The origin of HRF remains controversial and activated microglia or lipoprotein exudation are among the suspected causes [18], [19]. Growing evidence suggests that idiopathic epiretinal macular membranes present with lacunar structures with inflammatory or necrotic characteristics [20]. Based on the inflammatory exudates and leukocyte response in iERM eyes [21], [22], we believe that the appearance of HRFs in iERM eyes is closely related to microglia activation and inflammation. CD14 is a surface molecule of monocytic cells up-regulated after monocyte stimulation and involved in cellular activation [23]. Whereas CD163 is an endocytic receptor for haptoglobin–hemoglobin complexes and serves as a specific marker for M2 macrophages [24]. Both membrane-bound and soluble forms are involved in the activation of inflammatory responses. Based on this information, we focussed on the two inflammation-related cytokines. We divided the patients into two groups, those with and without HRF, and measured sCD163 and sCD14 concentrations in the vitreous fluid obtained from patients at baseline (Fig. 1). In the 43 eyes with iERM, the average vitreous level of sCD163 was 1381.9 ± 512.4 pg/ml in the 18 eyes with HRF and 814.4 ± 512.4 pg/ml in the 25 eyes without HRF. This difference was found to be statistically significant (*P* = 0.036, *Z*=-2.093) (Fig. 1A). Compared with the 95% CI, a total of eight eyes (8/18, 40%) showed presence of HRF but low expression of sCD163. Whereas four eyes (4/25, 16%) showed absence of HRF but increased expression of sCD163.

However, there was no significant difference in sCD14 concentrations between eyes with HRF (75.9 ± 99.9 ng/ml) and without (139.8 ± 164.7 ng/ml) HRF (*P* = 0.313, *Z*=-1.009) (Fig. 1B).

### Correlation between sCD163 levels and CMT changes from baseline

**Fig. 2:**
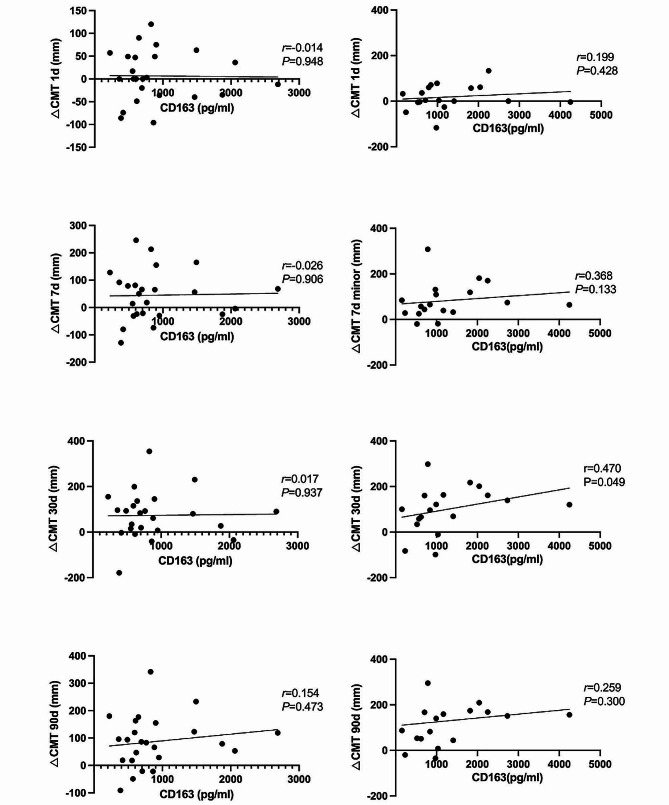
The correlation between the vitreous levels of sCD163 and △CMT at different follow-up times*(Left column: control group; Right column: DEX group)

Linear correlation analysis was conducted to investigate the relationship between sCD163 levels and CMT changes (Fig. 2). There was a significant positive correlation observed between the vitreous levels of sCD163 and △CMT at 1 month after surgery in the DEX group (*r* = 0.470, *P* = 0.049). Therefore, under DEX treatment, higher sCD163 levels in the vitreous fluid yielded greater CMT improvement. In contrast, in the control group, the vitreous levels of sCD163 were not significantly correlated with △CMT at any time during follow-up.

## Discussion

PPV with membrane peeling is the gold standard surgical treatment for patients with symptomatic ERM. It has been shown to result in improved visual acuity and morphology recovery in most patients with the condition [25], [26]. However, after PPV and ERM removal, visual and anatomical functional recovery is sometimes unsatisfactory. Residual ME remains an obstacle to complete visual function recovery in terms of decreased CMT and improved BCVA. Preoperatively, ME, caused by persistent attachment of the vitreous and inflammation, plays the principal role in the decline in visual acuity [27]. Mechanical surgical intervention can aggravate ME by damaging the blood-retinal barrier and promoting inflammatory exudates and leukocyte responses in the macular region. Growing evidence suggests that residual ME after PPV may have an inflammatory cause. Given this, some surgeons have been encouraged to use pharmacological treatments for persistent ME based on histological studies of steroid therapies.

Corticosteroids, which are powerful anti-inflammatory agents, have been found to inhibit the synthesis of vascular endothelial growth factor, prostaglandins, and many pro-inflammatory cytokines. However, when administered topically or locally, the drug levels in the vitreous are often suboptimal. Therefore, direct intravitreal injection is considered most effective method of achieving optimal drug levels in the vitreous [28]. The development of DEX has allowed for improved control over drug delivery potentially leading to lower rate of adverse events and reduced frequency of intraocular injections [29].

Several studies have reported on the efficacy of DEX in patients undergoing ERM peeling [6]–[10], and the conclusions are controversial. In a randomized controlled trial conducted by Guidi et al. [7], the effectiveness of intraoperative slow-release DEX implants in improving BCVA after 25-G vitrectomy and ERM removal was tested in patients diagnosed with idiopathic macular pucker. The results showed that after 6 months, both the control group and the group that received DEX implants demonstrated significant improvements in BCVA scores and reductions in foveal thickness. However, there were no significant differences observed between the two groups. Our study yielded similar results. The BCVA and CMT were not significantly different in the two groups. However, previous studies have not investigated the efficacy of DEX in different subgroups.

OCT biomarkers such as CME [15], MME [30], and DRIL [31], have been associated with deterioration of visual acuity in iERM after vitrectomy. Therefore, in our study, we conducted a qualitative analysis of OCT characteristics, including CMT, CME, MME, HRF, DRIL, and IS/OS continuity, to compare the ability of different anatomic biomarkers to predict postoperative outcomes after application of DEX implants in eyes with iERM. We identified HRF as a biomarker that could predict improved visual and anatomical outcomes after DEX implantation in eyes with iERM. Patients with HRF were more likely to be sensitive to Ozurdex implants than those without HRF.

Numerous studies have shown that HRF originates from activated microglial cells induced by inflammatory responses or lipoprotein exudation [19]. CD14 is expressed in microglia, monocytes, and macrophages [23], [32] and contributes to inflammatory responses activation when in its bound or soluble form [33]. Furthermore, the concentration of sCD163 concentration likely reflects the number of M2 macrophages and can be considered as a valuable biomarker for macrophage activation in different inflammatory diseases [34]. Therefore, we hypothesised that eyes with HRF would have elevated sCD163 and sCD14.

To test this hypothesis, we compared sCD163 and sCD14 concentrations in the vitreous fluid in patients with and without HRF. We found that the vitreous concentration of sCD163 was significantly higher in iERM patients with HRF than in those without HRF, whereas there was no significant difference in expression of sCD14. Accumulating evidence suggests that macrophages can be divided into two subgroups, M1 and M2 [35]. CD163 is an endocytic receptor for haptoglobin–hemoglobin complexes and serves as a specific marker for M2 macrophages [24]. Owing to ectodomain shedding, the extracellular part of CD163 is released as a soluble protein known as sCD163. sCD163 has emerged as a novel biomarker for diseases affecting macrophage function and monocyte/macrophage load in the body. Recent studies have demonstrated that sCD163 is a valuable biomarker for assessing macrophage activation in the retina [36].

Furthermore, we investigated the correlation between sCD163 levels and surgical outcomes. Our data demonstrated a correlation between vitreous levels of sCD163 and higher △CMT 1 month after surgery in the DEX group, indicating that vitreous fluid sCD163 level is a significant biomarker for predicting the efficacy of Ozurdex treatment. This time point is consistent with the findings of a pharmacokinetic study of DEX implants [37] which found an initially high rate of DEX release in the first 2 months after injection, followed by a decrease in release. This suggests an association between intraocular inflammation and the development or progression of certain iERM cases. DEX implants may be particularly effective in treating cases where ERM is secondary to inflammation which explain for the effectiveness of treatment in patients with HRF.

Howerver, despite the statistically different results, we observed outliers in each of the two groups for with or without HRF, implying that the correlation between HRF and sCD163 is not strong. The correlation should be confirmed by further follow-up studies with large sample sizes. Moreover, further immunohistologic studies using donor eyeballs from patients with iERM may be needed to obtain definitive answers as to whether HRF originates from activated microglia and whether elevated sCD163 in iERM is primarily due to activated microglia.

### Limitations

Although this study included a prospective assessment and offered meaningful results, it had some notable limitations. First, it was conducted with a small sample size and the follow-up period was relatively short. With a larger sample size, the grades of various OCT characteristics can be further subdivided to provide a more accurate prediction of prognosis. Therefore, future studies should include more patients to evaluate the correlation further. Second, the study design did not include a double-blinded population of patients and clinicians. This means that the results have a risk of bias, even if the researchers evaluated the data logical. Third, despite statistical differences, a certain number of outliers proved that the correlation between HRF and sCD163 was not strong. Regardless of our finding, imaging markers are still more likely to be used in clinical practice than molecular markers because of convenience and preoperative knowability.

## Conclusions

This study demonstrates that certain OCT biomarkers can help predict the response to DEX implants in patients with iERM. It has been shown that there is not generally a place for the use of DEX for iERM. Patients with HRF on OCT showed a better therapeutic response to DEX implants, which may guide the choice of clinical treatment options.

### Electronic supplementary material

Below is the link to the electronic supplementary material.

Supplementary Material 1

Supplementary Material 2

Supplementary Material 3

Supplementary Material 4

## Data Availability

All data and material are available from the corresponding author.

## Abbreviations

BCVA: best-corrected visual acuity
CMO: cystoid macular oedema
CMT: central macular thickness
DEX: dexamethasone
DRIL: disorganization of retinal inner layers
EIFL: ectopic inner foveal layer
iERM: idiopathic epiretinal membrane
HRF: hyperreflective foci
INL: inner nuclear layer
logMAR: log minimum angle of resolution
ME: macular oedema
MMO: microcysts macular oedema
OCT: optical coherence tomography
PPV: Pars plana vitrectomy
sCD14: soluble CD14
sCD163: soluble CD163
