# Supplementary figures and images for: Optical coherence tomography biomarkers as outcome predictors to guide dexamethasone implant use in patients with iERM: a randomized controlled trial
Source: BMC Ophthalmol. 2024 Apr 25;24:193. doi: 10.1186/s12886-024-03429-2 (PMC11044407; doi:10.1186/s12886-024-03429-2)

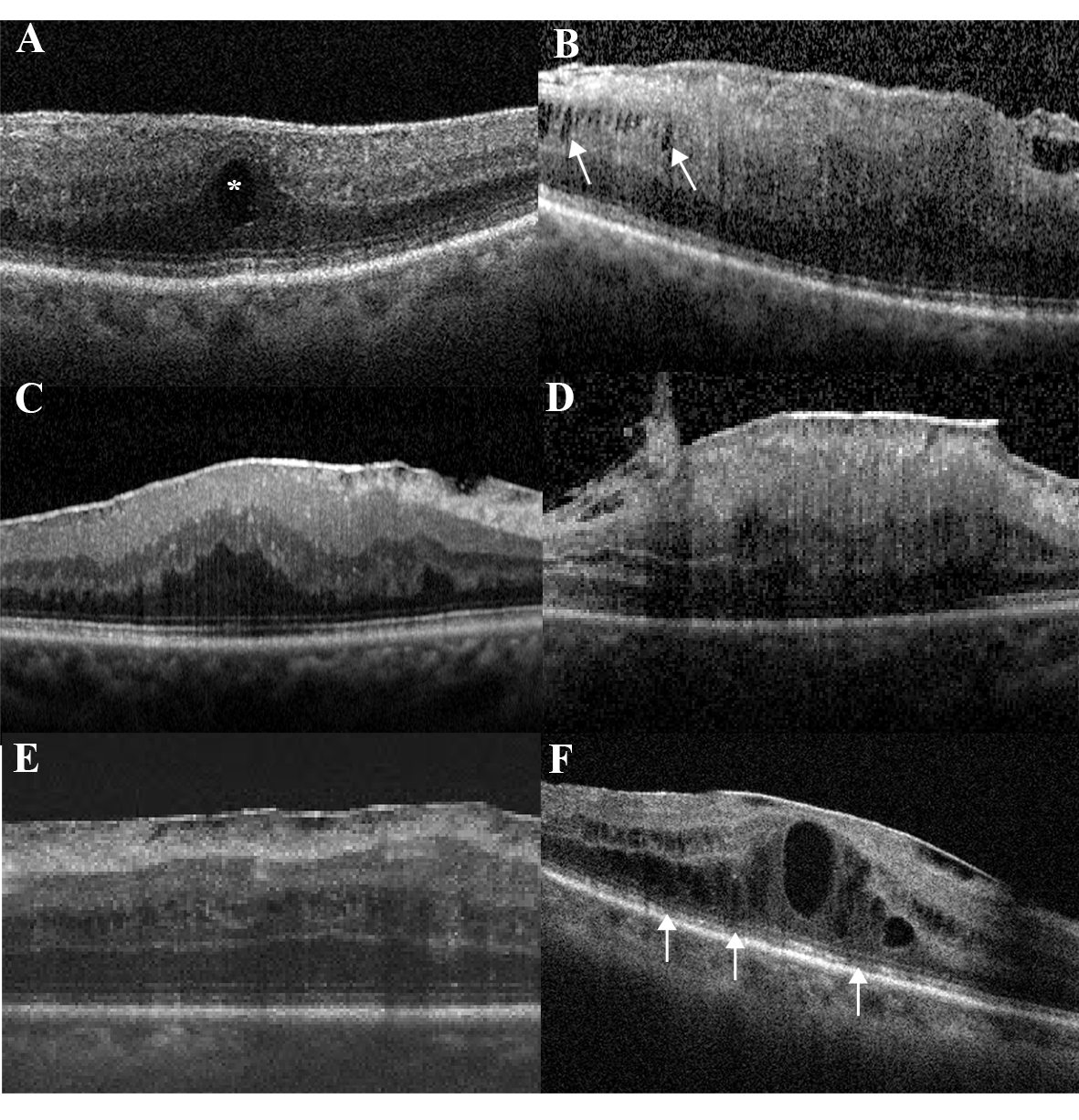

Supplement: Supplementary file 1 — Supplementary Material 1 [file 12886_2024_3429_MOESM1_ESM.tif]

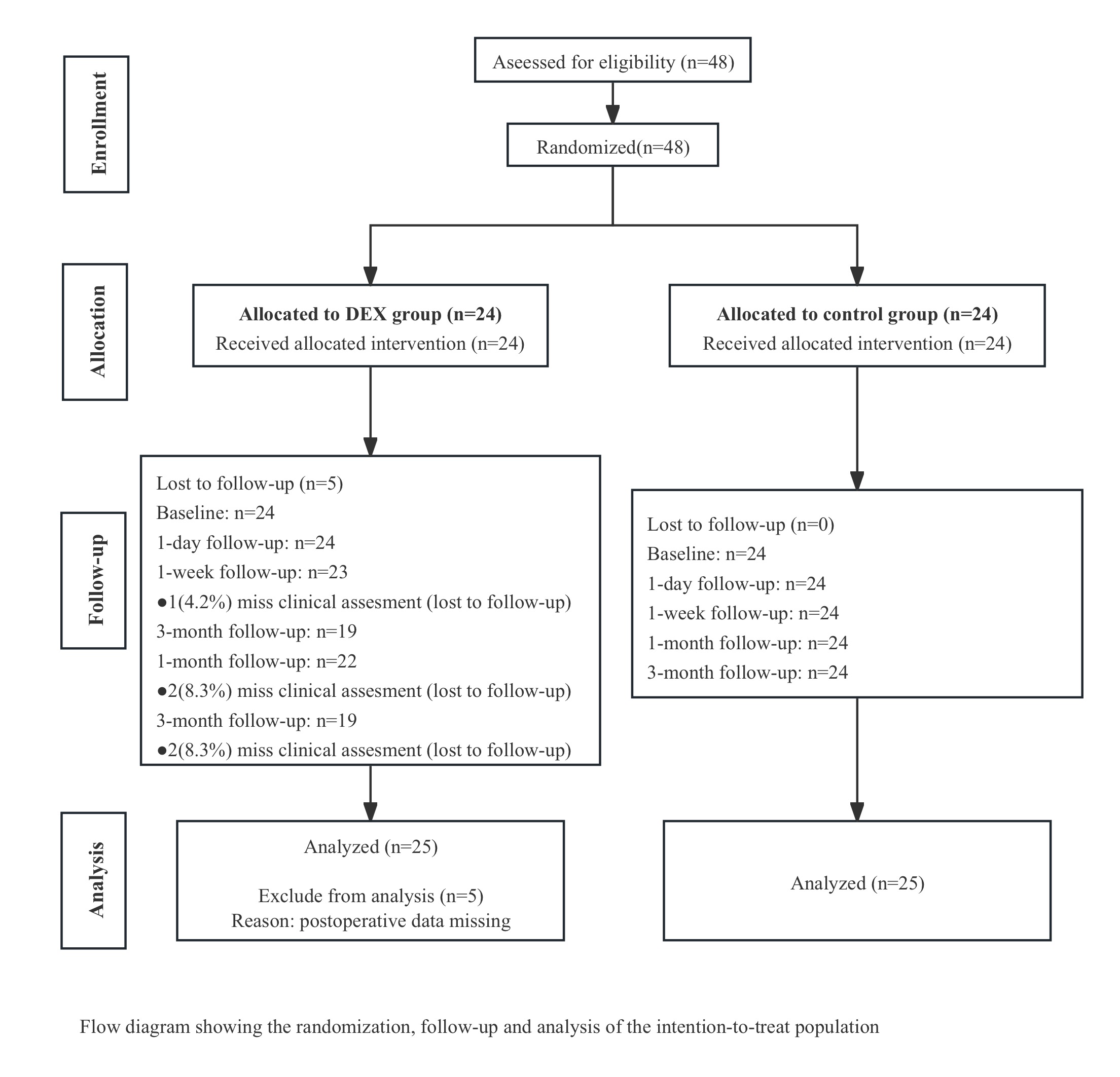

Supplement: Supplementary file 4 — Supplementary Material 4 [file 12886_2024_3429_MOESM4_ESM.jpg]
