# Supplementary material for: Optical coherence tomography biomarkers as outcome predictors to guide dexamethasone implant use in patients with iERM: a randomized controlled trial
Source: BMC Ophthalmol. 2024 Apr 25;24:193. doi: 10.1186/s12886-024-03429-2 (PMC11044407; doi:10.1186/s12886-024-03429-2)
Supplement: Supplementary file 2 — Supplementary Material 2 [file 12886_2024_3429_MOESM2_ESM.docx]

eFigure 1. Optical coherence tomography (OCT) measures.

A. Cystoid macular edema (*); B. Microcysts macular edema (arrow)

C. Hyperreflective foci; D. Disorganization of retinal inner layers; E. Continuous IS-OS layer; F. Disruption of the IS/OS junction (arrow).
