# Supplementary material for: Optical coherence tomography biomarkers as outcome predictors to guide dexamethasone implant use in patients with iERM: a randomized controlled trial
Source: BMC Ophthalmol. 2024 Apr 25;24:193. doi: 10.1186/s12886-024-03429-2 (PMC11044407; doi:10.1186/s12886-024-03429-2)
Supplement: Supplementary file 3 — Supplementary Material 3 [file 12886_2024_3429_MOESM3_ESM.docx]

**Supplement Table 1. Nonocular and Ocular Baseline Characteristics**

|  | DEX Group | Control Group | Value | *P* value |
| --- | --- | --- | --- | --- |
| No. of patients(eyes) | 19 | 24 |  |  |
| Male/female, n (%) | 6 (33.3%)/13 (66.7%) | 6(26.1%)/17(73.9%) | 1.03 | 0.303 |
| Age in yrs, mean (SD) | 65.50±5.07 | 68.95±7.14 | -1.85 | 0.071 |
| Laterality, OS n (%) | 7 (36.8%)/12 (63.2%) | 13 (54.2%)/11 (45.8%) | 1.131 | 0.359 |
| IOP, mean (SD) | 13.90±0.74 | 14.97±0.85 | -2.95 | 0.190 |
| BCVA (logMAR), Mean (SD) | 0.58 ±0.25 | 0.57 ±0.29 | 0.13 | 0.900 |
| CMT (mm), Mean (SD) | 444.8 ±109.0 | 449.0±82.8 | -0.14 | 0.891 |
| IOP (mmHg), Mean (SD) | 13.90±0.74 | 14.97±0.85 | 1.33 | 0.190 |
| Presence of CME, n (%) | 3 (15.8%)/16 (84.2%) | 7(29.2%)/17 (70.8%) | 0.65 | 0.515 |
| Presence of MME, n (%) | 9 (47.4%)/10 (52.6%) | 9 (37.5%)/15 (62.5%) | 0.51 | 0.613 |
| Presence of HPF, n (%) | 8 (42.1%)/11 (57.9) | 10(41.7%)/14 (58.3%) | 0.03 | 0.977 |
| Presence of EIFL, n (%) | 6 (33.3%)/13 (66.7%) | 7(29.2%)/17 (70.8%) | 0.17 | 0.864 |
| Presence of DRIL, n (%) | 9 (47.4%)/10 (52.6%) | 9 (37.5%)/15 (62.5%) | 0.65 | 0.515 |
| IS-OS continuity, n (%) | 5 (26.3%)/14(73.7%) | 7 (29.2%)/17 (70.8%) | 0.21 | 0.836 |
| BCVA = best-corrected visual acuity; CMT = center macular thickness; SD = standard deviation; CME = cystoid macular edema; MME = microcysts macular edema; HRF = hyperreflective foci; DRIL = disorganization of retinal inner layers; IS-OS = inner segment-outer segment; IOP = intraocular pressure; SD = standard deviation. | | | | |
